# Supplementary material for: A network approach to identify bioregions in the distribution of Mediterranean amphipods associated with Posidonia oceanica meadows
Source: PeerJ. 2019 Apr 23;7:e6786. doi: 10.7717/peerj.6786 (PMC6485239; doi:10.7717/peerj.6786)
Supplement: Supplemental Information 1 [file peerj-07-6786-s001.doc]

**Supplemental Materials & Methods S1**

**A network approach to identify bioregions in the distribution of Mediterranean amphipods associated with Posidonia oceanica meadows**

An extensive (meta) analysis of the current and past literature was conducted in order to extract all available information about the distribution of amphipods on *Posidonia oceanica* (L.) Delile, 1813 at Mediterranean basin level. We identified Summer (June to September) as the most frequent sampling period and a depth of 0.5-30 m as the most frequent range.

Unfortunately, no common sampling procedures were found in different studies, so that we focused on those able to sample (at least) motile organisms living in the leaf stratum. The final dataset was thus built by standardizing the information deriving form three main sampling procedures, briefly described below:

1. Air-lift sampling: is a technique originally described in Bussers et al. (1983) where an air-lift sampler with a 500-µm mesh size is used to sucked up at constant airflow (for at least 2 minutes) samples from the meadows. The sampling surface, with three replicates, is delimited by a 40×40 cm quadrant (Michel et al., 2010).
2. Hand-towel net: originally described in Russo et al. (1985), consists in a series of strokes to shake the leaves of *P. oceanica* from the basis, with the net (400-µm mesh size) held to form an angle of ca. 45° with the bottom (Bedini et al., 2011; Scipione et al., 1996).
3. Clumps collection: a technique where a quadrant of 30 cm side and 25 cm height was placed in each *Posidonia* meadow. Shoots are pulled up and collected in a bag with a net size of 0.3 mm, and species are subsequently sorted and identified in the lab.

In order to further homogenize the different datasets, we decided to retain only information about the presence/absence of amphipods on *Posidonia* *oceanica* meadows. Abundance data were only used to identify singletons (species found with a single individual in a single locality) that were then removed from the dataset.

Overall, the final dataset contains 28 localities, of which nine located in Tunisia, 18 in Italy (Tyrrhenian, Adriatic and Ionian Sea), two in Spain and one in Corsica (France) (see Fig.1 in the main text), for a total of 147 species (Table S1). Since data come from papers printed in different years and by different Authors, a possible problem concerning species nomenclature may raise. However, all the Authors used the reference taxonomic keys by Ruffo (1982-98) to identify the species recovered, so warranting for consistency in species identification. Also, in more recent works the Authors updated their nomenclature according to the World Register of Marine Species (WoRMS). It was therefore possible to track the taxonomic rearrangements occurred to the species’ nomenclature through WoRMS so that species names reported in Table S1 are fully updated (last access 05/03/2019).

**Table S1** – Species incidence matrix with reference literature listed for each species (see Supplemental References and Supplemental Data).

**Supplemental References**

Bedini R, Pertusati M, Batistini F, Piazzi L. 2011. Spatial and temporal variation of motile macro-invertebrate assemblages associated with *Posidonia oceanica* meadows. *Acta Adriatica* 52(2):201-214.

Bellisario B, Camisa F, Nascetti G, Lattanzi L, Cimmaruta R. 2016. Spatial and temporal variation of coastal mainland vs. insular amphipod assemblages on *Posidonia oceanica* meadows. *Marine Biodiversity* 46(2):355-363 DOI: 10.1007/s12526-015-0367-z.

Bussers JC et al. 1983. Description d’une suceuse à air comprimé économique et utilisable par un seul plongeur. *Cahiers de Biologie Marine* 24:215-217.

Camisa F, Abbattista C, Bellisario B, Angeletti D, Lattanzi L, Cimmaruta R. 2017. Seasonal variations of amphipod assemblages in a *Posidonia oceanica* (Linnaeus) Delile, 1813 meadow from the central Tyrrhenian Sea (Italy). *Crustaceana* 90:1319-1335 DOI: 10.1163/15685403-00003725.

Diviacco G. 1988. I Crostacei Anfipodi di alcune praterie di *Posidonia oceanica* pugliesi. *Thalassia Salentina* 18:131-139.

Michel L, Lepoint G, Dauby P, Sturaro N. 2010. Sampling methods for amphipods of *Posidonia oceanica* meadows: a comparative study. *Crustaceana*, 83:39-47 DOI: 10.1163/156854009X454630.

Ruffo S (ed.) 1982-1998. The Amphipoda of the Mediterranean. Parts 1-2-3-4. Mémories de l’Institut océanographique, Monaco, 13: I-XLIV, 1-959.

Russo GF et al. 1985. The hand-towed net method for direct sampling in *Posidonia oceanica* beds. *Rapport de la Commission Internationale Mer Méditerranée* 29:175-177.

Sánchez-Jerez P, [Carberá Cebrian](https://dialnet.unirioja.es/servlet/autor?codigo=1941641) C, [Ramos Esplá](https://dialnet.unirioja.es/servlet/autor?codigo=281387) AA. 2000. Influence of the structure of *Posidonia oceanica* meadows modified by bottom trawling on crustacean assemblages: comparison of amphipods and decapods. *Scientia Marina* 64(3):319-326.

Scipione MB et al. 1996. Vagile fauna of the leaf stratum of *Posidonia oceanica* and *Cymodocea nodosa* in the Mediterranean Sea. - Seagrass biology. Perth: The University of Western Australia, 249-260.

Scipione MB. 1998. Amphipod biodiversity in the foliar stratum of shallow-water

*Posidonia oceanica* beds in the Mediterranean Sea. In: Schram FR, von Vaupel Klein JC, eds. *Crustaceans and the biodiversity crisis*. *Proceedings of the Fourth International Crustacean Congress.* Leiden: Brill, 649-662.

Sturaro N, Lepoint G, Pérez-Perera A, Vermeulen S, Panzalis P, Navone A, Gobert S. 2015. Seagrass amphipod assemblages in a Mediterranean marine protected area: a multiscale approach. *Marine Ecology Progress Series* 506:175-192 DOI:

Sturaro N, Lepoint G, Vermeulen S, Gobert S. 2015. Multiscale variability of amphipod assemblages in *Posidonia oceanica* meadows. *Journal of Sea Research* 95:258-271 DOI: 10.1016/j.seares.2014.04.011.

Zakhama-Sraieb R, Ramzi Sghaier Y, Charfi-Cheikhrouha F. 2006. Is amphipod diversity related to the quality of *Posidonia oceanica* beds? *Biologia Marina Mediterranea* 13(4):174-180.

Zakhama-Sraieb R, Ramzi Sghaier Y, Charfi-Cheikhrouha F. 2011. Community structure of amphipods on shallow *Posidonia oceanica* meadows off Tunisian coasts. *Helgoland Marine Research* 65(2):203 DOI: 10.1007/s10152-010-0216-1.
